# Round the Hospitals

**Published:** 1918-06-15

**Authors:** 


### ROUND THE HOSPITALS.

The King has conferred the Military Medal, for great courage during the bombing of a clearing station, on Sister-in-Charge Kate Maxey, T.F.N.S., who, though severely wounded and in great pain, helped to rescue another sister who was fatally wounded; on Sister Dorothy Foster, R.R.C., T.F.N.S., for coolness in supervising the transfer of patients during bombing; and on Acting Sisters Mary Agatha Brown and Marie Dow Lutwick, both of Q.A.I.M.N.S. (R.), who were on duty with their matron when she was severely wounded, and a sister who was killed. Sister Lutwick crossed the open bomb-swept ground alone to get help. The King conferred also the Military Medal on Miss Lilian Furse, V.A.D., for her admirable coolness when in charge of a marquee on which a bomb fell injuring many patients.

Few distinctions will meet with more general acclaim in nursing circles than those conferred by His Majesty on the Matrons-in-Chief of Q.A.I.M.N.S. On Miss Becher, R.R.C., has rested some of the long, slow, laborious work of

**ROUND THE HOSPITALS—(*continued*).**

ensuring efficiency in her department of the Service, and the recognition she now receives in becoming Dame Grand Cross of the Order of the British Empire is a testimony all can understand. Her colleague Miss E. M. McCarthy, R.R.C., who directs the members of the Service on active duty in France and Flanders, becomes a Commander of the Order of the British Empire. She is endeared to countless nurses by her consideration for their safety and comfort, and the simplicity with which she faces hardship and danger at the call of duty. Her talent for organisation make her an inspiring example throughout her command.

SPEAKING in the House of Commons on June 10, Mr. Macpherson gave the following particulars of the recent hospital outrages in France:—During the period May 15 to June 1 hospitals have been bombed on seven occasions. Casualties to the nursing staff were five sisters killed and eleven sisters wounded. Total casualties, 941. The names of the sufferers are still withheld. We fear the number may be added to, for the disgraceful attacks on the wounded and on those who tend them still continue. A vivid account is given in the *Daily Telegraph* by Mr. Roland Hill, Canadian official correspondent, of the destruction of yet another Canadian hospital in an area never used for military purposes, the roofs being painted with great red crosses. One three-storeyed wing, 200 yards long, was cut in half by a huge bomb and then caught fire. The operation room was working at high pressure, as some badly wounded cases had just been brought in, and the entire staff, doctors, nurses and patients, were buried under an avalanche of debris. In a few minutes the whole operating section was a flaming tomb, and bursting tubes of ether and hydrogen added to the ghastliness of the scene. Two other surgical teams had mercifully just gone off to their midnight supper, or the casualty list of doctors and nurses would have been far heavier.

THE deliberate intention of the murderous airmen is exposed by the testimony of a matron and her nurses, who told Sir Arthur Stanley that the attacking air force used flares which lighted up everything. Not satisfied with dropping bombs they flew low and used machine guns on the escaping refugees. The bright spot in all these horrors is the behaviour of the nurses, who showed neither fear nor thought for themselves. The chaplain of one of the bombed hospitals told the King that during the attack the nurses refused to avail themselves of the protection which would have been afforded by getting under the beds. They did their utmost to cheer and comfort the patients, gave them pillows with which to cover their heads, and did for them whatever was possible. Many paid for their pluck and resource by cruel maiming.

MUCH sympathy is felt for the contingent of V.A.D. members, thirty-five in number, with Sister King in charge, belonging to the South African Military Nursing Service, who were wrecked in the *Kenilworth Castle*. Thirty-three of the party were safely landed, and owed their rescue largely to their coolness and presence of mind in danger. They quietly slipped on their life-belts and cloaks and awaited their turn for the boats, assisting the captain by calling out their names in turn when, owing to the pitch darkness, he failed to decipher the list. Miss Bolus and Miss Black were drowned. The body of the former has been recovered, and has been buried with military honours at Plymouth. Many of the rescued, including Sister King, were several hours in the water, and are suffering from the shock and the prolonged exposure. They are being well looked after by the South African Comforts Committee, and such as require it are receiving convalescent and other treatment, while all are enjoying an extra period of leave after their perilous adventure.

WE believe that the result of the College election will have caused considerable surprise in nursing circles. Ten previous members of the Council were re-elected, including three doctors, and one new medical member has been added to the list. This seems to show conclusively that nurses desire to have the aid of members of the medical profession in setting their house in order. The new members, Major-General Sir Berkeley Moynihan, C.B., M.S., F.R.C.S., and Miss Margaret Hogg, C.B.E., Matron of Guy's Hospital, will bring a further accession of influence to the College.

THE private Conference, on June 7, of Matrons past and present, members of the College, was of the greatest possible importance from the point of view of State Registration. There was an attendance of eighty matrons of general and special hospitals, and the chair was taken by Miss Sparshott, R.R.C., lady superintendent, Manchester Royal Infirmary. Amongst the speakers were Miss Gill, R.R.C., Royal Infirmary, Edinburgh; Miss Worsley, matron, Children's Infirmary, Edinburgh; Miss Lloyd Still, C.B.E., R.R.C.; Miss Cummins, Royal Infirmary, Liverpool; Miss Redl, matron, Brompton Hospital for Consumption; Miss Dalton, matron, Victoria Park Hospital for Diseases of the Chest; Miss Girdlestone, R.R.C., late matron, Crumpsall Infirmary, Manchester; Miss Lyons, matron, Children's Hospital, Hull; Miss Cox-Davies, R.R.C., matron, Royal Free Hospital; Miss Chaff, matron, Royal Cornwall Infirmary, and others. The question under discussion, "Special and Small Hospitals in Relation to Training-Schools," may be said to bristle with intricate problems. More than aught else, it is retarding the attainment of legislation, and it can only be settled on a satisfactory basis by close examination of actual conditions of training. Miss Lloyd

**ROUND THE HOSPITALS—(*continued*).**

Still, during the years she spent as matron at the Brompton Hospital for Consumption, had considerable experience in dealing with the problems of specialised training. It is probable that every kind of special hospital may need to adopt a system differing in some particulars from that of others. Hence the value of meeting in private conference where individual difficulties could be stated and individual suggestions considered. It is much that the College meeting should have brought this opportunity for taking counsel within reach of widely sundered authorities on the points involved. The lively interest roused by the subject was reflected in the animated and suggestive debate which took place on this occasion.

The papers read at the Conference on the Higher Education of Nurses and its Relation to Applicants for Training, which took place in the evening of June 6, after the College meeting, reached a very high level. The session was in charge of Miss Daunt, assistant matron of the Middlesex Hospital. Miss Gullan, sister-tutor at St. Thomas's Hospital, opened the discussion with a paper admirable in form and substance which we give in full on page 237. The spirit which animates the system of higher training, of which the appointment of a sister-tutor is the coping-stone, has never before been so forcibly set before the nursing world. "We needs must love the highest when we see it," and we believe not one of that crowded audience, many of whom contentedly stood in doorways to listen to the discussion, went away unconvinced of the larger possibilities which lie in the training of nurses. Even the old order, who dread the effect on nursing ideals of too many lectures, too much laboratory and note-taking work, must have been deeply impressed with Miss Gullan's reverence for "those thousand and one details, independent of ordered treatment, that minister to the patients' well-being and happiness, that grow out of example rather than precept, that form the intangible 'something' that distinguishes our British nurses all the world over." It is a paper which marks the coming of a new type of training, in which all that was of value in the old system will be treasured—perhaps set higher even than before.

**URGENT NOTICE.**

To ensure a copy of The Hospital regularly, readers must place a definite order with their Newsagent TO-DAY. After the middle of June, in consequence of the new Paper Restrictions Order, it will not be possible for Newsagents to stock copies for chance customers.

DO NOT DELAY. ORDER

**"THE HOSPITAL" TO-DAY**

and obtain your copy regularly EVERY THURSDAY.

The discussion which followed Miss Gullan's opening address centred round the question of education under some of its numerous aspects. There can be no disputing the fact that in this country up to the present time education, especially for women, has been a class distinction. A gulf still separates the girl whose father can keep her at school till she is eighteen, or at college for three or four years longer, and the girl who has to begin earning her own living at fifteen or sixteen. Miss Innes, matron of Leeds General Infirmary, laid much stress on the importance of "character" in the nurse, which she defined as that independence of mind which prepares one to face emergencies. She brought out the point, too often forgotten, that girls who surrender a leisurely home life for the duties of a probationer give some indication of vocation by that very act, and hence the majority are found to possess a genuine love for the work. But gifts of "character" are distributed pretty equally among all classes. It is only a question of weeding out the fit from those who are looking for a soft job, or take up nursing from motives lower than the best. There is plenty of room in the nursing profession for women not too well educated in the modern sense. Miss Amy Hughes, who argued that high ideals of nursing were not within the grasp of any but the educated, was controverted by Miss Wolseley Lewis, who exposed the need for middle-class nurses to do private work in middle-class households, where highly educated nurses would be less welcome. Happily, high ideals are often found flourishing in women whose educational chances have been very scanty, and are not the property of one class.

We note with pleasure that more than one speaker emphasised the need for extending to probationers during their time in hospital some of those opportunities for self-improvement which their life has hitherto failed to afford them. Miss Gullan's exposition of the sister-tutor's special work brought this aspect of the probationers' training into prominence, and Miss Clark, speaking on the art of training, declared that more time should be devoted to the revision of notes on lectures, because probationers were often deficient in expressing what they knew, and minute care in correction raised the general level of their education. It comes to this, that since all are agreed that there is an irreducible minimum of abstract matters necessary to be imparted to the probationer, and since the State up till now has not accepted responsibility for fitting all women to grasp these elements of science, it rests on the shoulders of the matrons and sisters in training-schools to bring the laggards into line, and to educate their probationers up to the required standard. Mr. Fisher is laying a foundation which in twenty years or so will relieve the hospitals of this strain; meantime the only road to success lies through Miss Gullan's methods.

But the ward sisters and their colleagues in the higher posts *must* be educated women. If training is to be a reality and not a mere drilling and bullying process, those who carry out the training must be teachers, with ability to impart what they themselves fully understand. Sister Wyles, of the Great Northern Central Hospital, ably expressed this point of view. In her opinion, and in that of other speakers, it is essential that sisters should be

**ROUND THE HOSPITALS—*(continued).***

chosen for their capacity to teach, and not be promoted from the nursing staff merely on their nursing abilities. When girls come from all classes to be trained they should find "an atmosphere of high endeavour, and duties sternly and rigidly carried out." In this way "the class from which probationers came would be of secondary importance." We believe this to be the key to nursing education. Miss Joseph, discussing this point, enforced the need for various grades of nurses within the fold, just as there are elementary school teachers, secondary school teachers, and university teachers, yet all belong to the teaching profession. "There should be no lowering of the standard of qualification for nurses, but standardised examinations should be held, to qualify for special departments; co-operation between the various training-schools could produce a linked system which would meet the needs of the community." It is a great ideal. In this as in all schemes for the more systematic education of nurses we come back to the educated ward sister, who, as Miss Bodley declared amidst applause, must be reckoned "the backbone of the nurse's training."

---

MISS BAILLIE, matron of the Royal Infirmary, Bristol, undoubtedly provided the sensation of the evening by her announcement, received with prolonged applause, that she had just succeeded in persuading her committee to grant a day off per week to each of her probationers. In a brief but telling speech she laid her finger on the great blot of the nursing profession, not pay, nor hard work, but over-long hours. This it was which made teachers in schools hesitate to recommend girls to take up nursing. Perhaps in years to come this eventful week for nurses will reckon Miss Baillie's announcement among its claims to remembrance. The Royal Infirmary at Bristol will for ever hold the distinction of inaugurating this great reform. It is the first British hospital in which nurses taste the common privilege of humanity, one day's rest in seven. We are confident that this privilege will rapidly extend as the benefits to be gained by it are demonstrated. Once eliminate the element of toil without rest from the hospital, and the strongest bar to the entry of educated women to the profession disappears. Miss Clark was able to announce that her committee at the Whipps Cross Hospital were in favour of granting one day in seven, but had been compelled, owing to war conditions, to postpone the inauguration of this reform. Miss Lloyd Still announced towards the end of the meeting that she was now receiving probationers at the age of twenty-one. With this reduction of the probationers' age in view we hail the new departure at the Bristol Royal Infirmary not merely as a most salutary but as a most necessary clause in the Real Nurse's Charter of Liberty.

WHILE matrons, sisters, and nurses talked, the Chairman, Sir Berkeley Moynihan, F.R.C.S., recently elected on the College Council, Sir Robert Jones, F.R.C.S., and Sir Arthur Stanley sat by, listened, and enjoyed. The chairman, in an interesting speech, recalled what many nurses present could remember, what shy, diffident, reticent children women of twenty-four used to be, and contrasted them with the present generation of women, who at twenty-one are both plastic and capable. He was very complimentary to the speakers. In no debates at medical societies had he heard "more sustained oratory or better opinions more admirably and concisely expressed." The cordial welcome he extended to the new reform at the Bristol Royal Infirmary is an earnest of the warm reception likely to be accorded to this step in other parts of England. "That was of good augury for the future, for all realised that the world was now in a molten condition: it was a time for new ideals, for the setting up of new standards, as well as for the sustaining of the ancient ideals which had served so well in the past." Further pleasant words from Sir Robert Jones and Sir Arthur Stanley brought the meeting to a close, and all must agree with him that the conference constitutes the best possible augury for the future success of the College.

---

THE Poor-Law Infirmary Matrons' Association recently passed a resolution in favour of the report of the Local Government Committee of the Ministry of Reconstruction. This has upset very much the *Poor-Law Officers' Journal*. It publishes an article, written in the curious English and the verbose style the journal uses, threatening the Association with the wrath of the Guardians and the National Association of Poor-Law Officers. This journal, which calls itself the organ of the Service, has published pages of adverse criticism of the report, but it cannot control its indignation when an important section of the Service dares to express a considered opinion with which it does not agree. The article would not be worthy of notice did it not call upon the National Association to interfere with the freedom of Poor-Law officers to express their views. What do the nurses who have joined the nursing section of this Association think of this appeal? This is not a question of the merits or demerits of the report of the Local Government Committee. It is a question of the right of the Poor-Law nursing profession to freedom of speech. What do the nurses who have joined the Association think of this attack on the independence of the Poor-Law nursing profession? They must take some notice of it. They must either acquiesce in the senile position in which the journal would place them, or they must assert the freedom of the profession to deal publicly with all matters affecting its interests.

---

☛ For The Higher Education of Nurses: Its Relation to Applicants for Training, see p. 237; Mother and Child in Scotland, p. 227; A Nurse's Charter of Liberty: Shylock's Argument Out of Court, p. 228; Matrons' and Sisters' Appointments, p. 236.

